# Initiating technology dependence to sustain a child’s life: a systematic review of reasons

**DOI:** 10.1136/medethics-2020-107099

**Published:** 2021-07-19

**Authors:** Denise Alexander, Mary Brigid Quirke, Jay Berry, Jessica Eustace-Cook, Piet Leroy, Kate Masterson, Martina Healy, Maria Brenner

**Affiliations:** 1 School of Nursing and Midwifery, Trinity College Dublin, Dublin, Ireland; 2 Division of General Pediatrics, Boston Children's Hospital, Boston, Massachusetts, USA; 3 Harvard Medical School, Harvard University, Boston, Massachusetts, USA; 4 The Library of Trinity College Dublin, Trinity College Dublin, Dublin, Ireland; 5 Pediatric Intensive Care Unit & Pediatric Procedural Sedation Unit, Maastricht UMC+, Maastricht, The Netherlands; 6 Paediatric Intensive Care, Our Lady's Hospital Crumlin, Crumlin, Ireland; 7 School of Medicine, Trinity College Dublin, Dublin, Ireland

**Keywords:** decision-making, technology/risk assessment, applied and professional ethics, quality/value of life/personhood, children

## Abstract

**Background:**

Decision-making in initiating life-sustaining health technology is complex and often conducted at time-critical junctures in clinical care. Many of these decisions have profound, often irreversible, consequences for the child and family, as well as potential benefits for functioning, health and quality of life. Yet little is known about what influences these decisions. A systematic review of reasoning identified the range of reasons clinicians give in the literature when initiating technology dependence in a child, and as a result helps determine the range of influences on these decisions.

**Methods:**

Medline, EMBASE, CINAHL, PsychINFO, Web of Science, ASSIA and Global Health Library databases were searched to identify all reasons given for the initiation of technology dependence in a child. Each reason was coded as a broad and narrow reason type, and whether it supported or rejected technology dependence.

**Results:**

53 relevant papers were retained from 1604 publications, containing 116 broad reason types and 383 narrow reason types. These were grouped into broad thematic categories: clinical factors, quality of life factors, moral imperatives and duty and personal values; and whether they supported, rejected or described the initiation of technology dependence. The majority were conceptual or discussion papers, less than a third were empirical studies. Most discussed neonates and focused on end-of-life care.

**Conclusions:**

There is a lack of empirical studies on this topic, scant knowledge about the experience of older children and their families in particular; and little written on choices made outside ‘end-of-life’ care. This review provides a sound basis for empirical research into the important influences on a child’s potential technology dependence.

## Introduction

The initiation of, or decision not to initiate, technology that will be required long-term to sustain a child’s life is extremely complex, often conducted at time-critical junctures in clinical care, such as when a child’s existing medical condition deteriorates, or is facing end-of-life care, generally in a paediatric critical care environment, such as paediatric or neonatal intensive care. Such decisions may have profound, and often irreversible, consequences for the child and for the child’s family. Factors that influence decisions around initiation may be inclusive of judgements about the best interests of the child and their future quality of life while supported by life-sustaining technology. However, the types of influences that may impact on decisions made by clinicians at this point-of-care delivery are largely unknown.^1 2^ This review deliberately focuses on clinicians’ influences, because of the lack of research into this group at this point-of-care delivery even though they are a key actor in the ultimate decision. This is not to deny the importance of patient and family decision-making, nor the value of shared decision-making. The aim of this review was not to evaluate decision-making frameworks for clinical practice, but to complete an in-depth academic exploration of the reasons and arguments in the literature for initiating or not initiating technology dependence in children made by clinicians. We have defined technology dependence required to sustain a child’s life in terms of a wide range of clinical technology to support biological functioning across a dependency continuum, for a range of clinical conditions.^3^ These are initiated within a complex biopsychosocial context and have wide-ranging sequelae for the child and family and for health and social care delivery. This is the approach to technology dependence taken for the purposes of this review.

To achieve this aim, we conducted a systematic review of reasoning, guided by the methodology outlined by Strech and Sofaer.^4^ This explores how influences on the initiation of technology dependence in acute paediatric healthcare have been, and are currently, conceptualised, in the scientific literature, and increases our understanding of the liminal space between the clinical diagnosis and eventual decision of the (nominally) primary decision-maker in the clinical team.

## Methods

The use of a systematic review of reasoning allows the application of a systematic approach to the argument-based literature of philosophical and empirical bioethics that discusses the issues of initiating life-sustaining technology. The review question is not an ethical question, but the factual question of which reasons have been given to address an ethical question. Thus, a review of reason allows us to identify the ethical questions and decisional elements surrounding technology initiation, from the point of view of clinicians. It achieves this by identifying the individual reasons given in the scientific literature, and assessing them in terms of whether each reason is regarded as ‘morally justifiable, impermissible, permissible or obligatory or for the view that a specific intervention should or need not be made’.^5^ Unlike a traditional systematic review, this review does not seek to settle the question of whether technology should or should not be initiated, nor does it provide guidance for decision-making in this context, as this would be impossible to predict. Instead, it presents an in-depth exploration of the arguments that exist in the literature, the reason being that a full set of reasons for or against the view in question is more useful than an inadequate ‘all things considered’ conclusion. This highlights the current gaps in these arguments in this fast-changing medical technology environment and can inform future empirical research around the initiation of technology dependence in children. This review of reasoning therefore asks: ‘Which reasons have been given for the initiation, or non-initiation, of technology dependence in a critical care environment to sustain a child’s life?’ A secondary question of the review is: ‘how have these reasons been used to argue that initiation of technology dependence in a child should or need not be undertaken?’

### Identifying the relevant literature

The search strategy was deliberately wide, and did not search specifically for ‘reasons’ or ‘reasoning’ because this would prevent the retrieval of many papers whose main purpose was to discuss reasoning, we were keen to ensure that all papers on the initiation of technology dependence were retrieved, so that we could capture reasoning that may be incidental to the main results of that paper. The search took place between March 2019 and June 2020. A three-strand search of the literature surrounding technology dependence in children was conducted with the aid of a specialist librarian. Seven key databases were identified to ensure a wide coverage of the literature. (EBSCO)Medline (1946–), CINAHL (1981–), PsycINFO (1990–), (Ovid) Embase (1966–), WHOLiS, Web of Science (1864–) and ASSIA (1970–). The databases were first scanned to identify appropriate index terms. A secondary keyword search string was then developed. This was populated using lists of synonyms and with input of keywords suggested by the research team, based on the key concepts of technology dependence; and physical disability, chronic illness and complex care needs. A combination of index terms and keyword search strings were used to create a systematic search. A sampler of the Medline search may be reviewed in the online supplemental material. Additional citation and bibliographic searches were conducted on all included studies to identify additional relevant studies for inclusion. All results were exported into Endnote for deduplication and then into Covidence for screening. Given the fact that this was the first time such a review was conducted on this topic the search had no limits on start and end dates.

10.1136/medethics-2020-107099.supp1 Supplementary data

### Study selection

Publications were included only if they met the inclusion criteria, these are explained in table 1.

**Table 1:** Inclusion criteria for the review

1	The paper includes a reason why technology dependence should be initiated or need not be initiated, and
2	The technology dependence is required to sustain a child’s life
3	The child referred to is cared for by healthcare professionals in a critical care environment
4	The publication is a peer-reviewed, published academic article or book, national-level report or working paper, PhD thesis, academic commentary or essay or case study and is available in English

**Figure 1:**
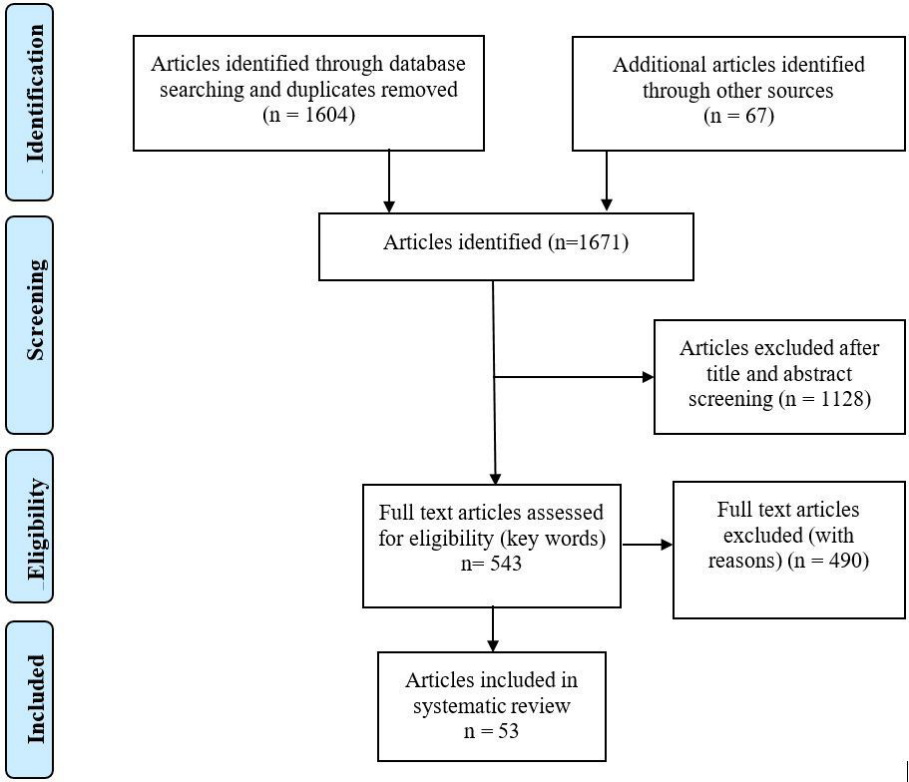
Data search and selection process.

We retrieved 1604 papers. After screening of titles and abstracts by two researchers, 1128 papers were discarded. A snowball search^6^ of the references of included papers, notes and bibliographies identified a further 67 relevant papers. A total of 543 papers were retained for full-text analysis to identify if reasons for initiation or non-initiation of technology dependence were contained in the text. We excluded 490 papers, primarily because they did not address the child population; or they did not discuss life-sustaining technology specifically, but addressed, for example cochlear implants, or wheelchairs which may aid life, but are not essential to sustain it. Papers were also rejected if they were not placed in the critical care environment, many papers focused on the challenges of living with technology-dependence at home, or at school or work. This process left 53 papers included in the review (figure 1).

#### Charting the data

Three tasks contributed to the charting of the data to minimise bias and also provide informative results. First, reasons were identified in the text by two researchers (DA and MB). Subsequently, each identified reason was coded by reason type, according to the passage in which the reason appeared; and finally, information about the context of the publication in which it appears (publication identification factors) were recorded.

Each reason identified was coded with a ‘broad’ and ‘narrow’ reason type.^3^ The codes were inducted from the reason itself and the text passage in which it was identified. A broad reason type gives a general description of the type of reason identified in a paper in one or two words, such as ‘responsibility’, ‘duty’ or ‘clinical need’. This gave a broad category for scrutiny. Each reason was also coded with a more detailed narrow reason type, which describes the context or intention of the reasoning, allowing greater interpretation of the reasons given. Examples of narrow reason types include ‘responsibility to preserve life’; ‘expectation of a good quality of life’ or ‘unable to survive without technology’. Two researchers coded a sample of 10 publications independently. Any discrepancies were identified, discussed and resolved between the two researchers, together with an objective third team member who was not part of this research exercise. This process enhanced the coding validity and reliability for the remaining papers, which were initially coded by one researcher. The codes were subsequently discussed at length by the rest of the research team, where any further discrepancies in interpretation were resolved.

In order to record subtle differences of meaning, a distinction was made between a mention of a reason expressed in a specific passage (a reason concept) often using the voice of the author from a direct reasoning from reported empirical evidence (reason mention), where the author reports directly the voice of the practitioner. Each reason was also coded in terms of whether it was in favour of the initiation of technology dependence, rejected technology dependence or whether the paper did not express a preference for or against initiation of technology dependence related to the reason expressed. We also coded, for each reason, whether the paper’s overall conclusion supported or rejected the reasoning, or whether the paper drew no strong conclusions about the reasons given.

Data about the publications and papers that contained the reasons were also recorded. We noted whether the paper (or the specific reason) discussed the initiation or non-initiation (including the withholding) of technology dependence; or was in the context of a discussion whether to withdraw technology from a child who was already technology dependent (eg, due to terminal diagnosis). We also noted if the reasoning for initiating or not initiating technology dependence was considered in the context of end-of-life care. We recorded, where possible, the age of the child. We noted the type of paper written (eg, review, empirical research results, discussion or essay) and the voice of the paper (generally a physician, nurse or parent). Finally, we recorded whether the paper was included in a peer-review journal, national report or book chapter as well as the field of study (such as medicine, nursing, law or bioethics), the date of publication, the setting and country of origin of the paper.

Patients were not involved in this research, other than in the identifying of patients’ or their advocates’ reasons for initiation or non-initiation of technology dependence.

## Results

A wide range of papers contributed to the review. The majority of papers focused on the ethical and legal discussions surrounding the use of technology to sustain a child’s life, without specifying the type of technology used. A small number specified the type of technology under scrutiny, the most common of which was long-term ventilation.

### Publication characteristics

**Figure 2:**
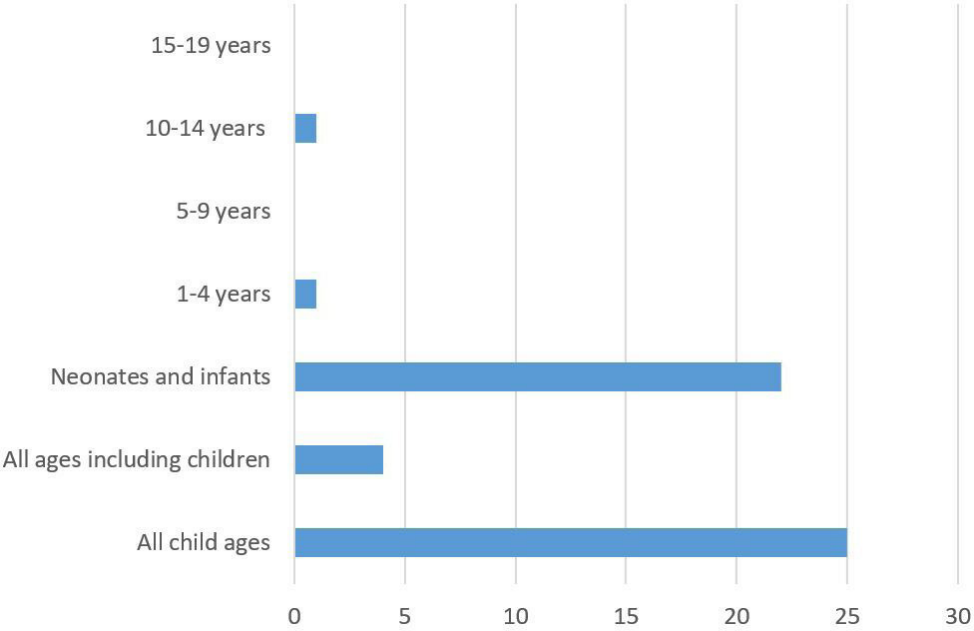
Ages of children who are the subjects of the reasons to initiate or not initiate technology dependence.

Fifty-three papers were included in the review. In the majority the reasons for or against initiation of technology, or for withdrawal of life-sustaining technology were identified as concepts described in the texts of reviews, or ethical essays (n=36). Thus, the reasons given were from the voice of the paper’s author, not directly from empirical evidence. Seventeen papers contained empirical data from interviews, surveys or focus groups of physicians. The majority of reasons shown were given by physicians, or reported as physician’s reasons for initiating or the non-initiation (or withdrawal) of technology dependence. Four papers also addressed nurses’ reasoning, or reasoning from the perspective of nursing; and two papers included reasons given by parents or foster parents of the child. Almost half of the publications discussed life-sustaining technology with regard to the neonatal or infant population (n=22). Some papers did not focus on children exclusively, but included all ages, with particular reference or a section on children, and discussions of children of all ages. No papers specifically dealt with children aged 5–9, although one mentioned a ‘preadolescent boy’ as the subject of a case study,^7^ and another includes a girl in this age range as a case study.^8^ No papers specifically referred to adolescents aged 15–19. Figure 2 summarises the patient age distribution of the identified papers.

Eighteen of the 53 papers in the review explored the initiation of technology dependence as part of end-of-life care. Seven papers investigated the initiation of care in the context of continuing this care longitudinally, outside the hospital setting. The remaining papers discussed the initiation and/or withdrawal of life-sustaining technology in a child in various disease, treatment or socioeconomic contexts.

**Figure 3:**
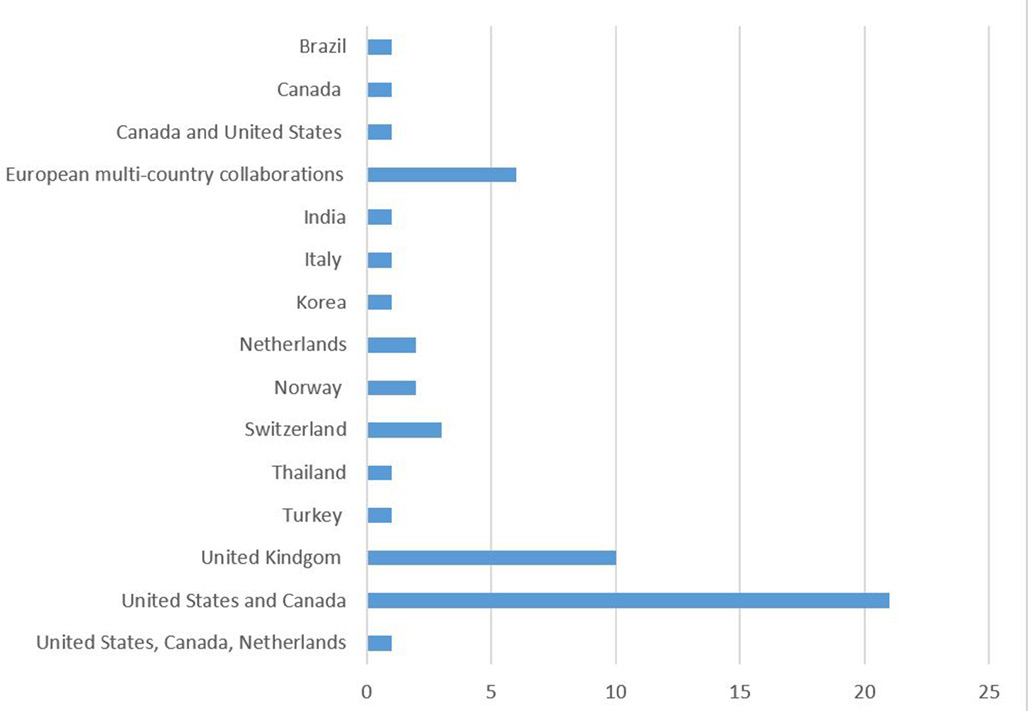
Distribution of reason types by country. European multi-country collaborations are: Luxembourg, Netherlands, Sweden, France, Germany, UK, Italy, Spain^13^; Italy, Spain, France, Germany, Netherlands, UK and Sweden^14^; Ireland / UK^15^;Germany / Switzerland^16^; Austria, Switzerland and Germany^17^; UK / Belgium^18^

Eleven papers were from sole authors, in the form of a review or providing an expert opinion about the ethical considerations involved. We found no systematic reviews into the issue. Two of the papers were in the form of guidelines or frameworks for practice, one of which was published in a peer-reviewed journal, and two were in the form of education pieces in peer-reviewed journals. Most of the papers were published in medical journals (n=37), nursing journals published eight; and another six were published in bioethical or legal journals. One paper was published in a health leadership journal, and two papers were published as book chapters. The publications included ranged in date from 1975 until 2020. The majority of the papers we identified came from developed countries, most commonly from the USA and Canada (see figure 3).

### Reason types and implications

We classified each identified reason in terms of whether it was in favour of initiating, not initiating technology dependence or reasons that were clearly related to technology dependence, but there could be no conclusion drawn as to whether they supported or rejected technology dependence (eg, a reason given as part of a philosophical discussion).

We identified 116 broad reason types; and 383 narrow reason types. None of the publications mentioned more than 32 broad or 41 narrow types of reason.

Most of the broad and narrow reasons given were not committed to being in favour of initiation nor against initiation of technology, although the reasons were directly related to the issue. For example, discussions around the initiation of ventilation for children at the end-of-life may be accompanied by reasons that do not specify whether an individual child should or should not receive ventilation. The vast majority of the papers supported the reasons given, as part of the wider discussion in the paper.

### Thematic breakdown of the reasons

#### Broad reason types

The reasons support, reject or describe the initiation of technology dependence. They were categorised into groups that emerged from the application of broad reason types in each individual paper, taking into account the context in which the reason was given. The groups identified were: ‘clinical factors’, ‘quality of life factors’, ‘moral imperatives and duty’ and ‘personal values’. Moral imperatives, for this purpose, are distinguished from personal factors and virtues because they are constructed by external norms of behaviour and codes of conduct.^9^ Each category contains a mixture of reasons describing internal factors, such as personal values, judgements and hopes; and reasons driven by external factors, such legal obligations, fear of repercussions or being guided by the wishes of parents or professionals, as expressed in the literature from which they were identified. Table 2 shows the broad reason types by category.

**Table 2:** Broad reason types identified in the review

	Clinical factors	Quality of life factors	Moral imperatives and duty	Personal factors and values
**Supporting technology initiation**	Benefit of treatment^19^; Best interest (of the child^17^; Clinical need^11 17 20–28^; Requirement to make a decision^14^; Prognosis^16^; Treatment^8 28–34^; Treatment decision^29^; Uncertainty^35^	Awareness of future consequences^20^; Future health^36 37^, Hope^38^; Hope for future technological improvement^20^; Possibilities of the technology^35^; Improve life expectancy^39^; Survivorship^40^; Well-being^28^	Altruism^38^; Coercion of physicians^41^; Communication 21 42; Decision-making^29^; Ethical conflicts^17^; Fear^13 19^; Human rights^23 24^; Information^31^; Requirement to share information^28^; Legal obligation^43^; Obligation^13 19 21 38^; Treatment resources^33^; Pressure^11^; Short-term outcome^26^	Anxiety^44^; Attitude towards value of life^35^; Being positive^35^; Experience maturity^35^; Hope for the future^37^; Purpose of role as physician^19^; Responsibility^23 26 35 45^; Values^17^; Wishes of parents or staff^46^
**Rejecting technology initiation**	Clinical decision^11^; Clinical need^8 11 13 21 28 45^; Clinical judgement^12 47^; End-of-life care^48^; Following guidelines^49^; Guidelines^17^; Intensive treatment^48^; Judgement of futility^14^; Prognosis^16 21 24 42^; Prognostication 22; Requirement to make a decision^14^; Treatment^8 27 29 34^; Treatment decision^26^	Quality of life 50; Unconsciousness of patient^43^; Well-being 28	Communication^15 24 42 51^; Corporate policy^49^; Decision-making^30^; Disputes and obstacles to good care^44^; Duty^12 52^; Ethics^12 29^ Fear^43^; Interpretation of policy^49^; Information provision^26 39^; Legal influence^47^; Medical principle^24^; Moral dilemmas^21^; Obligation to relieve suffering^19^; Obligation^53^; Purpose of role^19^; Responsibility^23 24 28 35 47 52^; Widespread belief^52^	Being familiar with patient or ward^13^; Compassion^19^; Cultural influences^49^; Distress^44^; Emotions^35^; Experience maturity^15 35^; Professional experience^13^; Professional objectivity 35; Religion^13^; Values 16 52
**Unclear whether supporting or rejecting technology initiation**	Advocating for child^23^; Best interests of child^15^; Care burden^54^; Care pathway^50^; Clinical need^11 39^; Decision-making^8 14 31 34^; Difficulty of prognosis^55^; End-of-life care^30 48^; Evaluation^16 56^; Evidence for prognosis^18^; Flexibility^24^; Guidelines^17^; Intensive treatment^48^; Judgement^24^; Prognosis 23 45 46 56; Prognostication^22^; Time pressure to act^15^; Treatment^8 28 29 32–34 48^; Treatment decision^8 26 32^; Uncertainty^35^	Longitudinal responsibility^26^ ; Outcomes^30 31 42 45^; Social value^7^ ; Socioeconomic differences^48^ ; Survival^54^ ; Well-being^28^	Beneficence^55^; Capacity and resources^18 50^; Communication^24 34 35 37 43 51 55 57^; Compromise^56^; Conflict 15; Contextual reasons^16^; Decision-making^8 29 30 32 57 58^; Dilemma^16^; Duty to act^59^; Duty to save life^15^; Ethical and philosophical meaning^7^; Ethical questions^26^; Family burden^16^; Fear^11 19 35 59^; Human rights^23^; Information 18 29–32 37 39 54 58; Justice^55^; Legal obligation^53^; Obligation^13 19 21 53^; Opportunity for discussion^13^; Overriding wishes of others 51; Parental decision-making^8 32^; Pressure^35^; Resource allocation^16^; Respect^24^; Role of physician^21^; Shared decision-making^17^; Sharing responsibility^19^; Sharing the decision^27^	Apprehension^19^; Assumptions^40^; Attitudes to decisions^8^; Compassion^59^; Conviction as in belief 7; Experience^28 35 45 56^; Feelings^35 39^; Grief^32^; Impartiality^15^; Information^60^; Information provision^26^; Openness^14^; Parental satisfaction^37^; Personal feelings^35^; Professional objectivity^35^; Responsibility^23 24 26 28 45 46 53 56 57 60 61^; Risk/danger^18 37^; Survivorship^19^; Values^16^

Reasons in the group describing clinical factors reflected a need to be impartial when making decisions—whether they supported or rejected technology initiation. Some reasons were more subjective, particularly in describing the potential benefits of treatment when supporting initiation of technology dependence, or describing the uncertainty of outcome after making a decision.

The theme that described quality of life factors included reasons that encompass a wider scope than the immediate medical needs of the patient, such as previous and future life, family well-being and mental as well as physical health. The reasons given for initiation of technology dependence in this category were primarily focused on the future, whether they concerned the initiation, rejection or description of initiating technology dependence.

The third group encompassed moral imperatives and a sense of duty informing the reasons given. This was the largest theme identified. Many of the moral or dutiful reasons for initiation were in fact negative in tone, for example describing the decision to initiate technology dependence because of fear of legal consequences or pressure from another party. The reasons given that had no clear direction for or against initiation of technology were primarily conceptual, describing ethical concepts of beneficence or duty to relieve family burdens. Reasons classified as personal factors encompassed elements of personal values, such as feelings of responsibility, positivity and attitudes; and also personal feelings such as anxiety, compassion or distress.

Some of the same reasons were used to argue for or against, or expressing no particular preference for the initiation of technology dependence. These included the best interests or future well-being of the child and also the need, or even the pressure, to follow guidelines or established procedures. Closely related to this are the reasons that describe the need for information provision and communication.

#### Narrow reason types

Three hundred and ninety reason types were identified in this review. The detail given by the narrow reasons, each of which accompanies a broad reason, gives further detail about the context and issues that inform the broad reason given. We identified different thematic headings depending on whether the reasons were given in support of, against or describing the initiation of technology dependence. The narrow reasons are shown in the online supplemental material.

**Figure 4:**
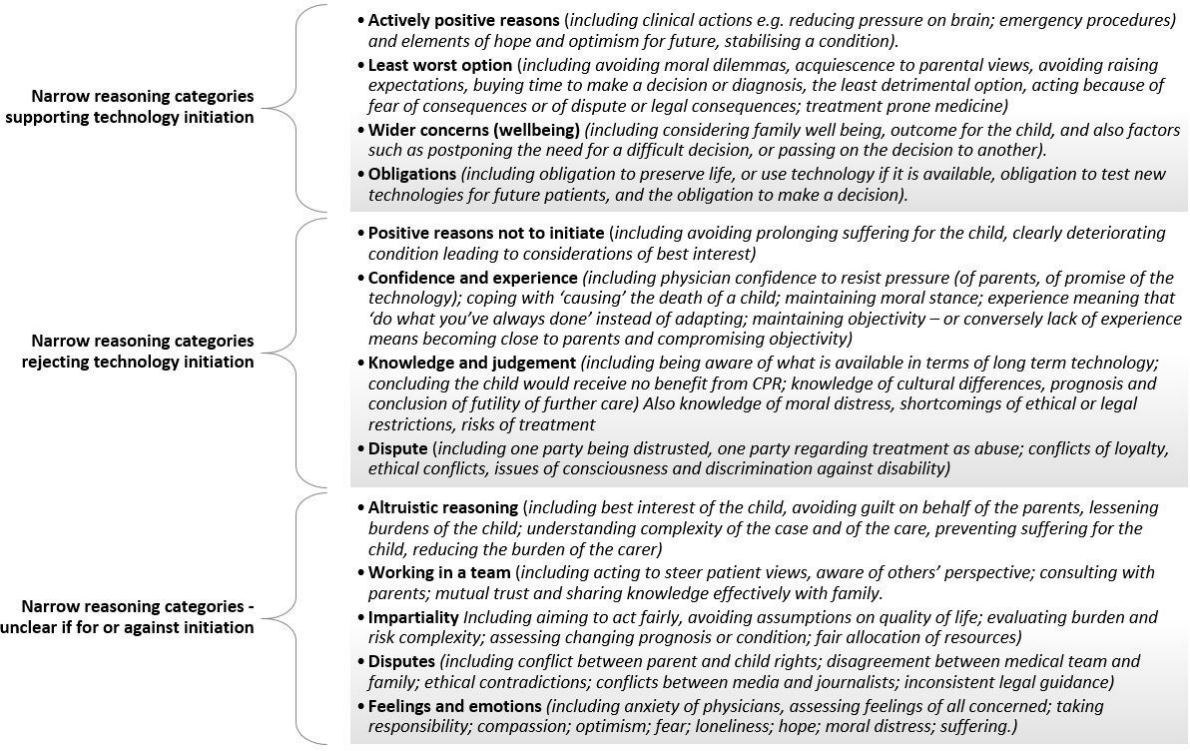
Themes identified in the narrow reason types.

Themes that emerge in the reasons supporting the initiation of technology dependence include actively positive reasons, aimed at improving the condition for the child, or preserving hope for the future. However, a theme of having to make a choice despite none being entirely satisfactory is also present, including buying time when a prognosis is not clear, obligation to initiate technology and the need to make the decision in the consideration of a wider context—such as future health, and family health and well-being. Figure 4 shows the themes identified.

Some of the reasons given not to initiate technology dependence also describe a number of different influences, including avoiding prolonging suffering for the child. Many reasons describe the experience and confidence of the decision-making physician—being able to make difficult decision, or to act in what is believed to be the child’s best interests despite the wishes of the child’s family. This encompasses themes of knowledge and professional judgement, and avoiding or effectively resolving disputes.

Reasons given without clear indication whether they supported or rejected technology dependence describe the issues that are perceived as important when technology dependence is a potential outcome. These reflect altruism and the need for effective communication in order for the involved parties to work effectively together. Reasons reflect the need for health professionals to take an impartial and wide-focused view on the situation, particularly when avoiding or resolving disputes. The need to take into account or recognise the influence of emotions, values and feelings also emerges from these reason types.

## Discussion

The aim of this review was to identify the reasons that have been given for the initiation or non-initiation of technology dependence in children. This allows an academic exploration to begin to understand this dynamic and ethically complex situation.

However, this exercise is of little value without a more in-depth examination of the implications of the reasons, and the pressure this puts on the complex ethical arguments that currently exist. Many of the reasons were used more than once, to justify more than one decision outcome, which demonstrates the complexity of the issue in respect of how the concerns and uniqueness of each situation are interpreted to make a decision. There are no ‘right answers’ to be found in life or in the literature with regard to initiating technology dependence in a child. Therefore, we identified reasons given for and against initiation of technology dependence and also reasons with no clear direction of intent given in the paper. By identifying the reasons that have been reported in the literature, this systematic review of reasoning allows a greater understanding of the implicit and explicit reasoning processes that influence the initiation of technology dependence.

### Range of publications

The papers identified in the review came from a range of sources: medical journals, nursing journals, law journals and publications from health management and bioethics. Most of the research or journal papers originated from developed countries, which was not unexpected given the subject matter of extremely sophisticated, and expensive, medical technology use. In addition, any cultural differences between technology dependence initiation in different countries would be an important avenue of research to explore, including the influence of the strength of religious beliefs in different country or cultural settings.

### Age of the children

In terms of the literature, it seems that most of the reasons given surrounding the initiation of technology dependence in children were relevant to neonates and younger children. There is scant literature specifically about the initiation of technology for older children, although there is increasing literature about children growing and living with technology dependence. We found no papers that discussed the initiation of technology for children aged 5–9 years, or for teenagers aged over 15 years. In addition, the focus on neonates as subjects of the initiation of technology dependence incorporates a number of ethical discussions about proxy decision-making, and about the concepts of quality of life for an infant; as well as navigating the potential dissonance in values and expectations of each of the decision-makers. Although these issues are present in all cases of children’s technology dependence (and arguably for adults too), neonates have no life experience to inform what their wishes should be. The grey areas of discussion and uncertainty are challenging in different ways for neonates than for other ages.

### Reasons for, against or about the initiation of technology dependence

Some of the reasons given reflect the physician’s attempts to retain an impartial role, by focusing on clinical factors, or using personal and professional experience to avoid making decisions informed by value-based or unfounded assumptions. However, many of the reasons identified suggest that value-based factors are important features of the process of initiating or rejecting technology dependence. Altruistic reasons such as alleviating guilt, doing the best for the child and taking responsibility feature in reasoning both for and against initiating dependence. The need for consideration of a child’s family and imparting information to the family to facilitate decision-making is important. But reasons surrounding this also suggest a degree of manipulation, such as imparting incomplete information—ostensibly for altruistic purposes, such as shouldering a burden of guilt and taking responsibility for decisions. This could be regarded as a form of paternalism, even if it is generally understood as an act of beneficence.

Some reasons were based on feelings such as anxiety and fear as influences on clinical judgement, as were emotions such as empathy, hope and compassion surrounding the child, family and other health professionals. Other aspects of non-clinical reasoning include obligation to act in certain ways, such as fear of prosecution despite feeling that not initiating or withdrawing technology dependence would be best for a terminally ill child. Resource issues were also given as reasons in some cases. Reasons may also be made that are aimed at preventing dispute, rather than being focused solely on the needs of the child. These may involve external influences, such as the threat of legal prosecution, or adverse personal consequences of a ‘trial by media’ or being in opposition to popular opinion.

Some of the reasoning given in the literature is actively positive, such as to relieve clinical symptoms, preventing adverse consequences of a disease or condition and achieving medical stability. However, the complexity of balancing risk and benefits for a child who may need technology to sustain life is challenging, and as a result, uncertainty is a common theme. Technology dependence may be initiated to gain more time to establish a diagnosis, prognosis or simply to postpone a difficult decision. Some decisions reflect legal or institutional pressure to treat a child, despite no clear outcome data to guide decisions. Some of the reasons given for initiating technology dependence were negative and reflected feelings of being coerced by the strong moral standpoints of other decision-makers, ethical conflicts and fear in the form of repercussions including legal consequences if technology is not used. Thus, the initiation of technology dependence is not always regarded as beneficial by the decision-maker but may be a source of moral distress if individuals feel that the child is subject to intrusive and painful treatment, or will never achieve subsequent good quality of life.^10^

In terms of the reasons given against the initiation of technology dependence, some of the reasons identified show how the ‘negative’ outcome of non-initiation is influenced by positive reasons. For example, when not initiating technology dependence is considered to be in the best interests of a severely ill child if death is inevitable and intervention would merely prolong discomfort or pain. In some of the narrow reason types, we see the effect of physician’s experience or confidence, such as the moral duty to relieve suffering, experience of outcomes and confidence to resist parental pressure, and the belief in a moral requirement to not provide futile care. Clinical factors, such as poor prognosis or terminal illness inform much of the reasoning not to initiate technology dependence. However, these are not incontrovertible as reflected in reason types such as coercion, obligation, ethical conflicts and fear, where in the papers the context shows that fear of legal repercussions or pressure from parents may also influence decisions. Some of the reasons given suggest the avoidance of dispute, or the input of an external factor instead of personal decisions, such as judgement of futility, following guidelines, apprehension and duty to act. Fear is an important stimulus for action, and appears in all three categories of reasoning, and in both broad and large reason types. Differences of opinion may lead to moral distress among healthcare professionals, as well as among parents, depending on the resulting decision.

Most of the reasons identified did not have a direct relationship with initiating or not initiating technology dependence, but were expressed to describe or discuss the surrounding issues. Most of the papers were essays or opinion pieces, where reasons were given as part of the author’s arguments. As a result, most of the reasoning is endorsed by the author. This may be because of a lack of empirical research, but also it is indicative of the challenging nature of the decisions. Some of the most contentious cases result in the need for external legal intervention to determine the best course of action,^11 12^ but even in these cases, decisions made depend on the circumstances that surround the clinical situation. This raises the question about the lack of recognition of all the voices of decision-making, including the voices of the physicians.

### Limitations

The majority of the reasons we identified were not found in papers that were specifically addressing the point of initiation of technology dependence in a child, but were identified in papers addressing issues related to this issue, such as the care of children with complex care needs, life-limiting conditions or papers addressing the ethics of life-sustaining technology. Therefore the reasons identified were not often discussed in terms of theoretical concepts that describe the arguments in which they were based.

Only English language papers were included. This may have excluded relevant papers that originate from non-English-speaking regions, such as in Africa, Asia, Europe or the Middle East. These countries may also provide different cultural perspectives to technology initiation, which could be identified in the reasons. In addition, the vast majority of the papers we identified were from North America, which suggests that there is a need to understand the voice of other parts of the world. We found that personal morals and values produce many reasons for decisions, and these are likely to be influenced by national cultures. Research data from all parts of the world, including Africa, the Americas, Asia, Australasia, Europe and the Middle East are likely to provide interesting data about influences and obligations concerning technology dependence in children.

The relevance of the systematic nature of the review is that a greater variety of reasons is likely to be identified through the systematic and extensive search process. However, it is potentially misleading to draw conclusions from the number of times a reason is mentioned in the literature. The review of reasoning is unable to critically analyse or weight the reasons given, principally because circumstances are different depending on the unique situation in which the reasoning is conducted. Also it is possible that there are more published literature that includes less contentious reasons, rather than those relating to controversial topics for example the influence of financial constraints. As a result, we did not count the number of times each reason appeared in the literature, because this is unlikely to represent the importance, or value, of any one reason and its influence on a decision.

Many of the papers retrieved in the literature search did not address the moment of technology initiation; this led to a considerable number of papers rejected for inclusion in the study at the stage of full-text reading. An increasing amount of literature describes children and families living with technology dependence rather than debating the point of its initiation. It is possible that this literature suggests a shift in focus from whether to initiate or not, towards the growing importance of future health and well-being.

Most of the reasons we identified were reason concepts rather than reason mentions. Any review depends on the quality of the studies on which it is based, and it is possible that the large number of papers that are discussion essays or expert opinion may constitute a bias in the results. A review of reasoning, however, does not attempt to critically appraise the reasons, but illustrates what reasons appear in the literature as influences on the initiation or non-initiation of technology dependence in children.

Most papers we identified discussed technology initiation in the context of end-of-life care. In these cases, technology may sustain life in a terminally ill individual. It is possible that there is a gap in our knowledge about initiation of technology dependence for children who are expected to live with technology dependence on a more long-term basis, because life-sustaining technology is only relatively recently, but increasingly, being used to this aim.

## Conclusions

This paper has explored *how* the influences on the initiation of technology dependence in acute paediatric healthcare have been conceptualised in the scientific literature. This was to identify which reasons have been given for the initiation or non-initiation of technology dependence in a critical care environment to sustain a child’s life and to examine how have these reasons been used to argue that initiation of technology dependence should, or need not, be undertaken. The wide variety of reasons we identified in the scientific literature extended beyond clinical factors, and involved moral, value-driven and personal influences on the child’s condition and future condition and potential treatment.

Relatively few papers focused specifically on the rationale for reasons given for action or non-action regarding technology dependence. This suggests that although this is an important point-of-care delivery, there has been scant specific exploration of the influences on decisions. The next phase of our research will begin to build on the findings from this review of reasoning, to focus on the influences on decisions in the liminal space between a child presenting with a life-limiting condition and the decision to initiate technology dependence. This is important in order to understand the interplay of conscious and unconscious influences, and the strength of these influences. Furthermore, research involving differing ages of the child is important to investigate if this alters the importance of influencing factors. Similarly, specific research in varying scenarios of technology dependence initiation, for example comparing influences on technology dependence in life-limiting situations, or as potential long-term support over many years is important to understand the range and importance of influencing factors. Increasing transparency at this point-of-care delivery allows the recognition and minimisation of bias and increases confidence in the process of accessing such care for patients, families and clinicians, potentially reducing disputes and reducing moral distress.

This research has provided a sound basis for greater understanding about access to care and the human relationship with technology. The number of children with complex needs who are subject to these debates and decisions is increasing. As a society, we struggle to articulate what is acceptable legally, ethically and medically, and importantly what constitutes a positive outcome in such complex cases. Transparency in the factors that influence care decisions will increase rigour, and make explicit influences, which are at present, largely unspoken.
