# Supplementary material for: Initiating technology dependence to sustain a child’s life: a systematic review of reasons
Source: J Med Ethics. 2021 Jul 19;48(12):1068–75. doi: 10.1136/medethics-2020-107099 (PMC9726963; doi:10.1136/medethics-2020-107099)
Supplement: Supplementary data [file medethics-2020-107099supp001.pdf]

SUPPLEMENTARY MATERIAL

Medline Search Sample:

Concept 1: technology dependence
Keywords: "technology depend*" OR "technology-depend*" OR "technological depend*" OR "technological-depend*" OR "screen depend*" OR "mobile depend*" OR "device depend*" OR "screen-depend*" OR "mobile-depend*" OR "device-depend*" OR "medical technol*" OR "dependen* on medical technol*" "technology relian*" OR "technolog*-relian*" OR "technological relian*" OR "screen relian*" OR "mobile relian*" OR "device relian*" OR "screen-relian*" OR "mobile-relian*" OR "device-relian*" OR "medical relian*" OR "relian* on medical technol*" OR "over*reliance in technolog*" OR "over*dependence on technolog*"

Concept 2: Physical disability OR chronic illness OR complex care OR complex medical care OR complex needs
Medline: (MH "Complex Care") OR (MH "Chronic Disease") OR (MH "Critical Illness") OR (MH "Disabled Persons+")

Keywords: "Physically Handicapped" OR "physical handicap*" OR "Physically Disab*" OR "Physical disability*" OR "Physically Challenged" OR "chronically ill" OR "chronic illness*" OR "chronic disease*" OR end-of-life OR "end of life" OR "life-limiting condition*" OR "life limiting condition*" OR life-threatening OR "life threatening" OR "terminal illness*" OR "life-limited" OR "life limited" OR life-sustaining OR "life sustaining" OR "sustaining life" OR "Life Limiting Illness*" OR "Life-Limiting Illness*" OR "Genetic Disease*" OR "Genetic Disorder*" OR "Genetic condition" OR "Inborn Genetic Disease*" OR "Single-Gene Defect*" OR "Single Gene Defect*" OR "Critical Illness*" OR "critically ill" OR "Catastrophic Illness*" OR "Multiple Chronic Conditions" OR "Multiple Chronic illnesses" OR "complex care" OR "complex caring" OR "complex nursing" OR "complex need*" OR "multifaceted need*" OR "multifaceted care" OR "complex medical need*" OR "complex medical care" OR "multiple needs" OR "multiple care" OR "medically fragile" OR "medically frail*" OR blind* OR "visual impair*" OR deaf* OR "hearing impair*" OR "mobility impair* OR "physical deficiency" OR "physical incapacity" OR "physically limited"

Technology	Medline
Technology	6,061
Phys Disab /Complex/Chronic	865,087
Combined	498
Peer Review	n/a
Total	498

#	Query	Limiters/Expanders	Last Run Via	Results
S6	S4 AND S5	Search modes - Boolean/Phrase	Interface - EBSCOhost Research Databases Search Screen - Advanced Search Database - MEDLINE	498
S5	AB (“technology depend*” OR “technology-depend*” OR “technological depend*” OR “technological-depend*” OR ”screen depend*” OR “mobile depend*” OR “device depend*” OR ”screen-depend*” OR “mobile-depend*” OR “device-depend*” OR “medical technol*” OR “dependen* on medical technol*” “technology relian*” OR “technolog*-relian*” OR “technological relian*” OR *” OR ”screen relian*” OR “mobile relian*” OR “device relian*” OR ”screen-relian*” OR “mobile-relian*” OR “device-relian*” OR “medical relian*” OR “relian* on medical technol*” OR ”over*reliance in technolog*” OR “over*dependence on technolog*”) OR TI (“technology depend*” OR “technology-depend*” OR “technological depend*” OR “technological-depend*” OR ”screen depend*” OR “mobile depend*” OR “device depend*” OR ”screen-depend*” OR “mobile-depend*” OR “device-depend*” OR “medical technol*” OR “dependen* on medical technol*” “technology relian*” OR “technolog*-relian*” OR “technological relian*” OR *” OR ”screen relian*” OR “mobile relian*” OR “device relian*” OR ”screen-relian*” OR “mobile-relian*” OR “device-relian*” OR “medical relian*” OR “relian* on medical technol*” OR	Search modes - Boolean/Phrase	Interface - EBSCOhost Research Databases Search Screen - Advanced Search Database - MEDLINE	6,061

	”over*reliance in technolog*” OR “over*dependence on technolog*”)			
S4	S1 OR S2 OR S3	Search modes - Boolean/Phrase	Interface - EBSCOhost Research Databases Search Screen - Advanced Search Database - MEDLINE	865,087
S3	AB (“Physically Handicapped” OR “physical handicap*” OR “Physically Disab*” OR “Physical disability*” OR “Physically Challenged” OR “chronically ill” OR “chronic illness*” OR “chronic disease*” OR end-of-life OR “end of life” OR “life-limiting condition*” OR “life limiting condition*” OR life-threatening OR “life threatening“ OR “terminal illness*” OR “life-limited” OR “life limited” OR life-sustaining OR “life sustaining” OR “sustaining life” OR “Life Limiting Illness*” OR “Life-Limiting Illness*” OR “Genetic Disease*” OR “Genetic Disorder*” OR “Genetic condition” OR “Inborn Genetic Disease*” OR “Single-Gene Defect*” OR “Single Gene Defect*” OR “Critical Illness*” OR “critically ill” OR “Catastrophic Illness*” OR “Multiple Chronic Conditions” OR “Multiple Chronic illnesses” OR “complex care” OR “complex caring” OR “complex nursing” OR “complex need*” OR “multifaceted need*” OR “multifaceted care” OR “complex medical need*” OR “complex medical care” OR “multiple needs” OR “multiple care” OR “medically fragile” OR “medically frail*” OR blind* OR “visual impair*” OR deaf* OR “hearing impair*” OR “mobility impair* OR “physical deficiency” OR “physical incapacity” OR “physically limited”) OR TI (“Physically Handicapped” OR “physical	Search modes - Boolean/Phrase	Interface - EBSCOhost Research Databases Search Screen - Advanced Search Database - MEDLINE	579,514

	handicap*" OR "Physically Disab*" OR "Physical disability*" OR "Physically Challenged" OR "chronically ill" OR "chronic illness*" OR "chronic disease*" OR end-of-life OR "end of life" OR "life-limiting condition*" OR "life limiting condition*" OR life-threatening OR "life threatening" OR "terminal illness*" OR "life-limited" OR "life limited" OR life-sustaining OR "life sustaining" OR "sustaining life" OR "Life Limiting Illness*" OR "Life-Limiting Illness*" OR "Genetic Disease*" OR "Genetic Disorder*" OR "Genetic condition" OR "Inborn Genetic Disease*" OR "Single-Gene Defect*" OR "Single Gene Defect*" OR "Critical Illness*" OR "critically ill" OR "Catastrophic Illness*" OR "Multiple Chronic Conditions" OR "Multiple Chronic illnesses" OR "complex care" OR "complex caring" OR "complex nursing" OR "complex need*" OR "multifaceted need*" OR "multifaceted care" OR "complex medical need*" OR "complex medical care" OR "multiple needs" OR "multiple care" OR "medically fragile" OR "medically frail*" OR blind* OR "visual impair*" OR deaf* OR "hearing impair*" OR "mobility impair* OR "physical deficiency" OR "physical incapacity" OR "physically limited")			
S2	(MH "Complex Care") OR (MH "Chronic Disease") OR (MH "Critical Illness")	Search modes - Boolean/Phrase	Interface - EBSCOhost Research Databases Search Screen - Advanced Search Database - MEDLINE	279,255
S1	(MH "Disabled Persons+")	Search modes - Boolean/Phrase	Interface - EBSCOhost Research Databases	61,659

			Search Screen - Advanced Search Database - MEDLINE	

Narrow reason types identified by the review

**Narrow reasoning categories for initiation**	**Narrow reasoning categories against initiation**	**Narrow reasoning categories - unclear if for or against initiation**
Actively positive reasons	*Positive reasons not to initiate*	*Altruistic reasoning*
Action to reduce pressure on the brain[28]	Alleviate moral dilemmas[22]	Accurate information and wellbeing[29]
Acute situations[25]	Avoiding distress for the child[11]	Alleviate burden of guilt[25, 29, 61]
Acute treatment to avoid death[32]	Avoiding prolongation of suffering[13]	Alleviate burden on parents[34]
Benefit to patient[38]	Acute deterioration of respiratory condition[15]	Authority to make decisions[56]
Child unable to meet nutritional needs orally[28]	Best interest and open discussion[25]	Best interest of the child[11, 27, 29, 42]
Doctor-centred[27]	Best interests of the child[29]	Burdens on family, family context[33]
Emergency situation [26, 28]	Best interests, avoiding intensive futile care[47]	Burden on parents[55]
Established diagnosis and treatment[17]	Caring responsibility[27]	Burden of treatment at home[37]
Feeding tube for survival[23]	Cultural factors supporting child death at home[34]	Capacity of services and resources[19]
Hope for a miraculous or unexpected good outcome[16]	Due consideration to value of continuing life sustaining support[24]	Carer well-being[29]
Hope/ expectation of good quality of life[33]	Family, caring responsibility[29]	Child well being[29]
Improve or ensure respiration[28]	Future quality of life, life expectancy[30]	Child and family under stress[39]
Improved child health[29]	Improve quality of life, preserving remaining life[15]	Control family's uncertainty through selective communication[14]
Life prolonging attitude[21]	Parental insight into child's prognosis[34]	Decisions based on rang of conditions, experience and prognosis[19]
Long term outcome[27]	Poor quality of life on technology[38]	Doubt about parent understanding of consequences[21]
Medical stability of the child[18]	Providing supportive comfort care[8]	Empathy[27]
Need for respiratory support[23]	Quality of life would be compromised by treatment[8]	Empathy from professionals[27]
Perceived as safe and expected treatment[37]	Respect patient or parent's wishes[51]	Evaluating patient future quality of life[33]
Preserving hope[21]	Responsibility to prevent needless pain[25]	
Recovery or bridge to transplantation[34]		

Role of healing[14]	Sensitive communication and respect[47]	Evaluating what family can bear[33]
Technology to improve survival[39]	Withdrawal of care as ethical in futile cases[17]	Family well-being[29]
	Withdrawal of technology dependence[32]	Fear of frightening the parents[21]
Least worst option		Greater prospects of survival but living with substantial disability[37]
Acquiescence to parent views[31]	*Confidence and experience*	Lack of home care nurses to provide home support[54]
Active euthanasia (attitude negative)[40]	Acceptability[31]	Life-sustaining and prevention of suffering - holistic outcomes[11]
Avoid raising expectations that create a moral dilemma[22]	Confidence to resist pressure[21]	Morbidity post-technology dependence[38]
Buy time to make a reasonable diagnosis[22]	Causing the death of the child[21]	Outcome likely to be poor[32]
Child well-being[29]	Closeness to patient prevents objectivity[21]	Overriding parental decisions based on child welfare[47]
Choice of treatment reflects resources[37]	Distinction between acts and omissions[51]	Perceived inability to provide good quality of life[32]
Choosing the least detrimental alternative (slight negative tone)[14]	Duty to preserve life and health[51]	Poor outcome[32]
Complex decision making[32]	Duty to the dying and to the sick is different[12]	Quality of life[34]
Continue short-term invasive therapy[43]	Easier to limit LST in young children[33]	Responsiveness to patient needs[61]
Disagreement with relatives (attitude negative)[40]	Experience of outcomes[46]	Risk to child and family health[39]
Fear of charges of homicide, abuse or neglect[14]	Favouring the status quo[51]	Taking burden from parents[31]
Keeping alive for benefit of others (author against this reason)[24]	Length of professional experience[40]	Tensions between sanctity of life and quality of life[19]
Legal consequences (attitude negative)[40]	Moral duty to uphold ethics despite the consequences[51]	Uncertain prognosis and what is best interests of child[42]
Legal consequences influencing action [13]	Moral obligation to relieve suffering[14]	Uncertainty of future improvement[41]
Legal obligation[14, 40, 44]	Morbidity, device malfunction and poor quality of life[38]	Unclear prognosis[46]
Maximum therapeutic care despite physician opinion[34]	No confidence to withdraw treatment if survival possible[46]	Understanding complexity of case[14]
Neonatal resuscitation unclear prognosis[17]	No parental support[30]	Understanding future consequences[29]
Not withdrawing care without prognosis[17]	Non-acquiescence to parent views[31]	Values of physician - best interests of the child[33]
Ongoing dependence[41]	Not required to provide futile care[52]	

Over optimism and appearing not to give up[19]	Physicians not to provide services that are futile[49]	
Parental hope of miracle recovery[34]	Poor prognosis and no benefit of treatment[32]	*Working in a team*
Treatment[27]	Responsibility to minimise harm and maximise benefits in a clinical and wider sense[51]	Act to steer patient views[14]
Treatment prone medicine[21]	Risks to home caring abilities[29]	Appropriate language used to describe unclear options[56]
Treatment to allow clear assessment[42]	Taking action (withdrawing care)[21]	Aware of parent or patient perspective[53]
Uncertain prognosis[21]	Taking burden from parents[31]	Burdensome to talk about death[44]
	Technology only prolongs death[22]	Child's ability to communicate views[8]
Wider concerns (wellbeing)	Technology dependence not beneficial[50]	Consensus decision making[58]
Family well being[29]		Consideration of advance directives[60]
Future quality of life, future consciousness[33]	*Knowledge and judgement*	Consultation with family but clinical team make final decision[25]
Influence of personal beliefs[17]	Awareness of home mechanical ventilation in adults influencing paediatricians[15]	Counselling families of all possibilities[32]
Minimise risk of long term morbidity[19]	Child receive no benefit from CPR[25]	Difference in perception of well being[38]
Outcome for the child[21]	Correcting nocturnal or proven (diurnal) ventilatory failure[15]	Difficult treatment decisions[32]
Post investigation[26]	Cultural differences as influences on ethical differences[48]	Effective communication[29]
Postponing or passing on responsibility[21]	Cultural variation in 'position statements' of viability[48]	Effective communication[11]
Pressurized by parents to initiate[46]	Disability future quality of life[49]	Effective communication for the child and parent[57]
Prolonging treatment opposed to active euthanasia[24]	Fatal or terminal illness – prognosis[40]	Empathy and appreciation for parental decision making[59]
Survival but increased morbidity[35]	Futile care[49]	Ensure full understanding of options[56]
Treatment covered by health insurance[37]	Futile treatment withdrawn[25]	Family assessment of child's condition[33]
	Futility of care[33]	Family caring responsibility[29]
Obligations	Futility of further treatment[12]	Family understanding of clinical need[39]
Experience of physician to make decisions[8]	Future consequences for patient views / views of	Future family wellbeing[45]
Obligation to preserve life[14]		Indirect involvement of parents[31]
Obligation to use the technology if it is there[16]		
Others will benefit from testing the technology in this case[16]		

Parental desire for treatment[8]<br><br>Pressure of public expectation of technology's capabilities[11]<br><br>Sole decision making[21]	patient[42]<br><br>Individual unit approach to decision making[48]<br><br>Ineffective treatment[29]<br><br>Lack of confidence in ethics[21]<br><br>Lack of community resources leads to intensive treatment at end of life[50]<br><br>Legal and moral considerations[33]<br><br>Legal support[47]<br><br>Miserable and unhappy[21]<br><br>Moral distress[27]<br><br>National differences in determining futility[31]<br><br>Need for agreement[21]<br><br>Need to relieve life-threatening symptom[29]<br><br>No chances of survival[30]<br><br>Non elective ventilation[15]<br><br>Non-curative treatment[29]<br><br>Obstacles to transition home[45]<br><br>Over-pessimistic[23]<br><br>Poor future quality of life[11]<br><br>Poor neurological prognosis[11, 22, 25, 40]<br><br>Providing nutrition and hydration only[24]<br><br>Regret reported by parents who opted for tracheostomy[36]<br><br>Severity of prognosis[8]<br><br>Socio-economic and cultural differences of parents[48]<br><br>Treatment risks [29]	Information about diagnosis and prognosis[54]<br><br>Information for parents for options at end of life[34]<br><br>Lack of evidence as guidance for parents[54]<br><br>Lessons learned from family[29]<br><br>Long-term caring requirements for the child[39]<br><br>Mutual trust and shared knowledge[25]<br><br>No opportunity for parental involvement[34]<br><br>Opinions of other professionals[21]<br><br>Parents as information receivers[34]<br><br>Parents as significant decision makers[34]<br><br>Parental decision based on medical advice[47]<br><br>Parental involvement[31]<br><br>Parents may prefer a form of paternalism[36]<br><br>Patient autonomy[29]<br><br>Patient's future ability to interact with others[7]<br><br>Presence of hospital ethics committee[40]<br><br>Physicians as information providers[34]<br><br>Potential for conflict[32]<br><br>Psychological support to parents[38]<br><br>Reassurance from colleagues, experts, ethical committee[57]<br><br>Receptive to parental / family needs and views[29]<br><br>Repeated opportunities for imparting information[36]<br><br>Respect for other members of clinical team[25]<br><br>Risk of catastrophic event at home[54]<br><br>Risk of social isolation in the long term[39]

	Updated guidelines support Withholding or withdrawing care [40]<br><br>*Dispute*<br>Being distrusted, treatment as abuse [13]<br>Courts discussing values of respect for life [49]<br>Discrimination against disability [49]<br>Facilitating parental change of mind once accepting of terminal diagnosis [34]<br>Families reluctant to consent [39]<br>Futility as a means of reclaiming lost decision-making power [12]<br>Loyalty conflicts [46]<br>Parental refusal of consent [11]<br>Parents' wishes are legally classed as euthanasia [44]<br>Persistent vegetative state legal possibility to discontinue treatment [44]<br>Reducing fear of legal consequences [17]<br>Resisting parental request [30]<br>Secrecy of acts/ euthanasia [14]	Risk of unilateral decision making[32]<br>Sharing decisions to empower families[28]<br>Shared discussions leading to decisions[32]<br>Sharing the decision[8]<br>Supportive and empathetic information provision[29]<br>Support to make decisions slowly[39]<br>Time to make best decisions[29]<br>Timely discussion before a child requires technology dependence[32]<br>Timing of information to inform decisions[50]<br>Understanding how to communicate information[59]<br>Willingness to involve parents in decisions[17]<br><br>*Impartiality*<br>Aiming to act fairly[53]<br>Assessment of quality of life[23]<br>Assumptions based on quality of life[41]<br>Availability of resources[33]<br>Avoid value-based or unfounded assumptions on quality of life[42]<br>Avoiding close relationships with parents[46]<br>Certainty of outcome[24]<br>Changing prognosis/child's situation[25]<br>Complex evaluation of burden and risk[42]<br>Confidence in decision[21]<br>Cost and complexity of care[54]

		Criteria for withholding or withdrawing treatment[34]<br>Culturally appropriate communication[53]<br>Death expected or survival unacceptable quality of life[24]<br>Decision based on diagnosis, prognosis and quality of life [24]<br>Delegating effective care[61]<br>Differences between neonates and older children[8]<br>Economic resource costs[33]<br>Economic and labour costs[45]<br>Estimation of quality of life[23]<br>Excluding information believed to be irrelevant[43]<br>Financial or economic impact[29]<br>Future challenges in diagnosis[28]<br>Future morbidity[28]<br>Future outcomes[57]<br>Future quality of life[57]<br>Global fair allocation of resources[53]<br>Guidelines influence decision making[17]<br>Importance of quality of life on decision making[24]<br>Increased survival but also morbidity[55]<br>Information reflecting linguistic and cultural needs[50]<br>Institutional treatment and caring[29]<br>Judgement of benefit or risk of treatment[25]<br>Legal obligation[14, 29, 40]<br>Longitudinal outcome[27]

		Non curative treatment[29]<br>No difference in mortality between those who do and don't rely on technology[36]<br>Objectivity hiding parts of the truth[21]<br>Obligation to be responsible for care at home[61]<br>Outcomes[27, 53]<br>Overtreatment[21]<br>Physician responsible for final decision[25]<br>Potential for consciousness[24]<br>Power/trust in technology[29]<br>Prolong life[61]<br>Rationing of resources[58]<br>Realistic assessment of problem[14]<br>Responsibility[27]<br>Responsibility for outcomes[61]<br>Responsibility to avoid bias or judgement about quality of life[42]<br>Responsibility to do the least harm[53]<br>Right to determine how skills are used[57]<br>Role is to preserve life[22]<br>Technology potentially incompatible with child growth and development[38]<br>Treatment associated with benefits and burdens[36]<br>Treatment related factors - invasiveness, complications, reversibility, life expectance, functioning, quality of life, psychology[33]<br>Uncertain or inaccurate prognosis[21, 43]

		Uncertain outcomes[27]<br>Uncertain prognosis[8]<br>Using facts to determine moral decision[43]<br>Wider concept of best interest of the child[25]<br>Worth the economic costs[27]<br><br>*Disputes*<br>Answers to disputes are empirically based[7]<br>Complex ethical and legal challenges[8]<br>Conflict between child rights and parent rights[46]<br>Conflict between hospital and community care[45]<br>Conflict in decision making[36]<br>Conflict with media/ journalists[46]<br>Conflict with nurses[46]<br>Difficult to communicate because of health care requirement to treat[44]<br>Dull the need to reappraise actions[43]<br>Fear of tracheostomy[39]<br>Inadequacy of outcome measures to inform prognosis[19]<br>Increased conflict in adults[8]<br>Inconsistent legal requirements international[52]<br>Information is understood differently by both parties (parents and clinicians)[55]<br>Intensive treatment at end of life[50]<br>Lack of availability of skilled home nursing care[37]

		Lack of consensus between physicians[8]<br>Loyalty conflicts[46]<br>Media spotlight[58]<br>Not using information in decision[54]<br>Obstacles to transition home[45]<br>Parents sidelined due to complex clinical situation[35]<br>Paternalism of information provision[24]<br>Physician / family disagreement[29]<br>Physician doubt about parent motivation[21]<br>Potential litigation[11]<br>Potential for racial bias in care provision[50]<br>Pressure from parents[21]<br>Public nature of discussion dictates action[14]<br>Recommending a treatment they would not want for themselves[27]<br>Risk of paternalism[56]<br>Socioeconomic disparity at the end of life[50]<br>Undisclosed fears of prosecution[14]<br><br>*Feelings and emotions*<br>Anxiety influencing clinical judgement[42]<br>Anxiety and fear of the entire process (family)[39]<br>Assessing feelings[61]<br>Assuming responsibility for risk of the decision[57]<br>Attitudes influenced by personal and psychological factors[8]

		Awareness / consciousness[24]<br>Compassion for life 'slippery concept'[58]<br>Considerations of quality of life and values[8]<br>Decisional regret after tracheostomy[36]<br>Emotions and discomfort[33]<br>Experience, optimism, patient subjective experience[33]<br>Fear and anxiety, loneliness[21]<br>Fear of media criticism[21]<br>Fear of mistakes[21]<br>Fear of prolonging suffering[46]<br>Good and bad[61]<br>Hesitation in withholding and withdrawing[33]<br>Hope[27]<br>Increased emotional input - humour, anger, animation[7]<br>Lack of importance of prognosis to experienced parents[35]<br>Less regret because technology was better than expected[36]<br>Moral distress[27]<br>More experience greater fear of negative media attention[21]<br>Narratives of being ‘good parents’ with no regrets[19]<br>Need to do something[36]<br>Not allowing anxiety to influence clinical decision[42]<br>Overwhelming power of technology[22]

		Personal agony[14]<br>Personal convictions and values[8]<br>Personal factors and experiences influencing decisions[8]<br>Presence of hope[29]<br>Relieve suffering (ambivalent)[61]<br>Relieve suffering[58]<br>Regret[36]<br>Stigmatisation[29]<br>Suffering at end of life[35]<br>Transparent and honest[29]
